# Report of the annual meeting of the Society for Medical Education in the German speaking countries, virtual from the ETH Zürich 2021

**DOI:** 10.3205/zma001536

**Published:** 2022-04-14

**Authors:** Sissel Guttormsen, Andrea Lörwald, Felicitas Wagner, Eva Hennel, Kai P. Schnabel, Rainer Weber, Jörg Goldhahn, Sören Huwendiek

**Affiliations:** 1 Universität Bern, Institut für Medizinische Lehre, Bern, Switzerland; 2 Universität Bern, Institut für Medizinische Lehre, Abteilung für Assessment und Evaluation, Bern, Switzerland; 3 Universität Zürich, Medizinische Fakultät, Dekanat, Zürich, Switzerland; 4 ETH Zürich, Departement Gesundheitswissenschaften und Technologie, Zürich, Switzerland

## Introduction

After the successful GMA Annual Meeting 2016 in Bern [1], we were *“innovative together”* for a second time in 2021. The GMA Annual Meeting 2021 was jointly organized by medical education and healthcare institutions in the Zurich area and the Medical Faculty of Bern, namely: University of Zurich, Medical Faculty; Swiss Federal Institute of Technology (ETH) Zurich; Careum Bildungsmanagement; Zurich University of Applied Sciences; University of Lucerne, Department of Health Sciences and Medicine; and Medical Faculty of Bern, with the Institute for Medical Education. After the GMA 2020 had to be postponed due to the Corona Pandemic, it was decided to hold the 2021 meeting online to ensure a higher planning reliability and to ensure to definitely be able to organise the GMA Annual Meeting again. *Innovatively*, we have thus implemented the first ever online annual meeting of the GMA. 

## Keynotes

The conference presidents Sissel Guttormsen (Bern) and Rainer Weber (Zurich) opened the conference on September 16, 2021 under the motto “Together innovative 2021: Learning in (public) healthcare” live from the studio in Zurich together with Thorsten Schäfer, chairman of the GMA, Sören Huwendiek, chairman of the scientific advisory board and Jörg Goldhahn, responsible for the technical implementation at the ETH. 

We continued the idea of the Tandem Keynotes from 2016: shorter contributions but multiple perspectives. Thus, 553 participants were able to enjoy the first tandem keynote on *Digital Transformation: potential, strategies and ethical reflections* from the comfort of their own homes. In the contribution *Shaping the transformation – potential and strategies towards a Blended University* by Oliver Janoschka, the potential of digitalization was first highlighted. Then Vanessa Rampton took us deeper into philosophy and ethics in this field with her presentation *Ethical reflection and medicine's digital transformation.*

With the second keynote we wanted to explore the keynote format even further, so a digital panel discussion on *Educational frameworks: drivers for curricular innovation*, was implemented. Martina Kadmon, Ursina Baumgartner, Wolfgang Prodinger, Mathieu Nendaz, Christoph Berendonk and Cécile Ledergerber highlighted the perspectives on this from Germany, Austria and Switzerland.

The third keynote, in tandem format, by Roman Hari and Anne-Sophie Blunier provoked and enlightened with the title *Peer teaching: more than cheap labour?* With their many years of experience from two perspectives as lecturers and as peer teachers, and their own research on the topic, they quickly convinced us of the many benefits, some unexpected, of peer teaching for both learners and young teachers. 

The final keynote addressed the topic of *Networking and interprofessionalism*. It was moderated as a panel discussion by Sylvia Kaap-Fröhlich. Together with René Ballnus, Irina Cichon and Andreas Gerber-Grote, opportunities and risks of interprofessional education in Germany, Switzerland and Sweden were discussed on the basis of educational actors, educational locations, educational experiences and necessary resources.

## Innovative formats in the programme

In addition to the “classic” contribution formats such as online short presentations, posters and workshops, the innovative formats introduced in 2016 were also offered. There were “Flipped Contributions”, in which the audience could prepare for the individual topics in a targeted manner, so that the presentation time during the conference could be used for a more intensive exchange and discussion. During the “demos”, products such as learning tools or various software could be presented. A highlight were the “fringe” sessions, in which serious topics were approached in a special way or reported in a whacky way. Last but not least, a brand-new feature was the opportunity for conference participants to submit “new/different formats” and, if accepted, to try them out. 

All formats were used diligently. From a total of 396 submitted abstracts from two submission rounds in 2020 and 2021, the scientific advisory board selected the conference papers based on the two-rounds peer review. The conference abstracts are published on the German Medical Science (GMS) portal and can be viewed at [https://www.egms.de/en/meetings/gma2021/]. 

In the end, the conference participants were offered a rich program, in which they could choose from 47 workshops, 35 short communication sessions, 7 “flipped contributions”, 4 “demos”, 2 “fringe” sessions and 8 “other/new formats” (e.g. science slam, poster karaoke, an exhibition on medical comics, learning to do research with Asterix, a BarCamp session, etc.) in addition to four mentioned keynote contributions. For the 173 posters, a new virtual poster evening was created. Those who could not attend a session in the very compact program from Thursday noon to Friday evening (September 16-17, 2021) had for the first time the opportunity to view the presentations afterwards via video.

During the conference, most of the 25 scientific committees and 8 sections met virtually to exchange content and formal aspects. Activities such as symposia and workshops on committee-specific activities were planned. 

The scientific advisory board of the GMA and the editors of the Journal for Medical Education met to further develop the journal and the scientific objectives of the society.

In the board meeting, the relationship of the GMA to the individual countries was discussed and then in the general meeting the mission of the board and the objectives of the professional society were discussed and a new board was elected.

## Awards

As every year, outstanding achievements were honoured with GMA awards. The GMS-JME Publication Award went to Petra Zimmermann and Martina Kadmon for their paper *Standardized examinees – development of a new instrument to assess factors influencing OSCE results and for use in examiner training*. 

The GMA Award for Innovative Teaching Project Ideas went to Pauline Wittekind, Berenike Hasagic, Likas Stüker, Janna Czichowski, Alina Mager with their project *MedScape room*.

The GMA project prize for the Further Development of Teaching went to Fabian Dupont, Aline Salzmann, Catherine Bopp, Sara Volz-Willems, Johannes Jäger with the project *Competence-oriented blended learning in general medicine, in cooperation with Amboss and the IMPP*. 

The GMA conference award for the best innovative contribution (Fringe category) went to Bertram Otto, who made us laugh and think with his contribution *Flipped digitization:* *the power of board games in medical education of digital natives*.

The GMA conference award for the best innovative contribution (non-Fringe category) went to Jan Matthes for his contribution Poster Karaoke or* “I don't know what's on my poster until my counterpart tells me”*.

## Retrospection and evaluation

One might assume that a virtual annual meeting is easier to organize than a face-to-face meeting, since many aspects, such as the exhibition, the physical registration of the participants or the catering are omitted. The opposite was the case and there were countless new tasks to manage. The live broadcast from the ETH Zurich video studio, the provision of the digital conference platform iStage and the ongoing technical support for the participants are just a few examples. We had to shorten the program from three days to two due to limited attention and tolerance to actively engage online for several hours per day. The GMA Annual Meeting has a tradition of open exchange with discussions during the various events. Even if we have all gained a lot of experience with online events in the meantime, the lively face-to-face “feeling” did not quite translate into the online format. Furthermore, the spontaneous contacts, exchanging ideas during the breaks, stopping at a poster and talking to the author, having a beer in the evening, exploring Zurich, ... could not be realized in the virtual space as in real life. Nevertheless, we looked for the best possible approximation, even if concessions had to be made.

245 out of 553 participants took part in the evaluation of the GMA Annual Meeting 2021. Despite the many challenges, most participants were satisfied (30%), very satisfied (10.4%) or rather satisfied (27.9%) with the event. A similar picture emerged in feedback regarding the online implementation of the conference, with the user-friendliness of the platform being the biggest point of criticism. 

The program was rated positively overall (28.9% rather satisfied, 37.5% satisfied and 16.4% very satisfied). The “workshops” (28.6% very satisfied and 39% satisfied) and the “other/new formats” (25% very satisfied and 49% satisfied) scored particularly well. The variety of topics was also highlighted as a particularly good aspect.

Regarding the organization of the conference, the ratings were mixed. 12.0% were not at all satisfied, 17.0% not satisfied, 21.2% rather not satisfied, 17.4% rather satisfied, 23.2% satisfied and 9.1% were very satisfied. The poor ratings seem to be mainly related to lack of or late information before and during the meeting.

Many participants would have appreciated an easier and more intensive exchange with the other conference visitors. In the future, the majority of participants would like to see a hybrid format for the GMA Annual Conference (51%). 40% would welcome a face-to-face event, 9% have spoken out in favour of holding the event online again.

## Outlook

We look forward to exploring the topic of “Form and Function - Digitization for and in Teaching” in more depth again face-to-face at the next GMA Annual Meeting, September 15-17, 2022 in Halle (Saale), Germany. More information can be found at [https://www.gma2022.de]. 

## Acknowledgements

We would like to thank everyone who made the first virtual GMA Annual Meeting 2021 possible, especially the CH Planning Group and the Scientific Advisory Board with Christoph Berendonk, Thomas Bucher, Andreas Gerber-Grote, Sylvia Kaap-Fröhlich, Mathieu Nendaz and Christian Schirlo. We would also like to thank Thorsten Schäfer and the entire GMA board for their support and the trust they have placed in us.

## Competing interests

The authors declare that they have no competing interests. 
